# Critically short telomeres and toxicity of chemotherapy in early breast cancer

**DOI:** 10.18632/oncotarget.15592

**Published:** 2017-02-21

**Authors:** Miguel Quintela-Fandino, Nora Soberon, Ana Lluch, Luis Manso, Isabel Calvo, Javier Cortes, Fernando Moreno-Antón, Miguel Gil-Gil, Noelia Martinez-Jánez, Antonio Gonzalez-Martin, Encarna Adrover, Raquel de Andres, Gemma Viñas, Antonio Llombart-Cussac, Emilio Alba, Silvana Mouron, Juan Guerra, Begoña Bermejo, Esther Zamora, Jose Angel García-Saenz, Sonia Pernas Simon, Eva Carrasco, María José Escudero, Ruth Campo, Ramón Colomer, Maria A Blasco

**Affiliations:** ^1^ Breast Cancer Clinical Research Unit, CNIO-Spanish National Cancer Research Center, Madrid, Spain; ^2^ Telomeres and Telomerase Group, Molecular Oncology Programme, CNIO-Spanish National Cancer Research Center, Madrid, Spain; ^3^ Medical Oncology Department, Hospital Clinico Universitario, Valencia, Spain; ^4^ Medical Oncology Department, Hospital 12 de Octubre, Madrid, Spain; ^5^ Medical Oncology Department, Hospital de Montepríncipe, Madrid, Spain; ^6^ Medical Oncology Department, Hospital Vall d´Hebron, Barcelona, Spain; ^7^ Medical Oncology Department, Hospital Ramón y Cajal, Madrid, Spain; ^8^ Medical Oncology Department, Hospital Clinico San Carlos, Madrid, Spain; ^9^ Medical Oncology Department, Institut Catala d'Oncologia-IDIBELL, L'Hospitalet de Llobregat, Barcelona, Spain; ^10^ Medical Oncology Department, MD Anderson Cancer Center, Madrid, Spain; ^11^ Medical Oncology Department, Hospital General de Albacete, Albacete, Spain; ^12^ Medical Oncology Department, Hospital Lozano Blesa, Zaragoza, Spain; ^13^ Medical Oncology Department, Institut Catala d'Oncologia-Hospital Dr. Josep Trueta, Girona, Spain; ^14^ Medical Oncology Department, Hospital Arnau de Vilanova, Valencia, Spain; ^15^ Medical Oncology Department, Hospital Clínico Universitario Virgen de la Victoria, Málaga, Spain; ^16^ Medical Oncology Department, Hospital Universitario de Fuenlabrada, Fuenlabrada, Spain; ^17^ GEICAM, Madrid, Spain; ^18^ Medical Oncology Department, Hospital Universitario La Princesa, Madrid, Spain

**Keywords:** telomere length, critically short telomeres, breast cancer, weekly paclitaxel, toxicity

## Abstract

Cumulative toxicity from weekly paclitaxel (myalgia, peripheral neuropathy, fatigue) compromises long-term administration. Preclinical data suggest that the burden of critically short telomeres (< 3 kilobases, CSTs), but not average telomere length by itself, accounts for limited tissue renewal and turnover capacity. The impact of this parameter (which can be modified with different therapies) in chemotherapy-derived toxicity has not been studied.

Blood from 115 treatment-naive patients from a clinical trial in early HER2-negative breast cancer that received weekly paclitaxel (80 mg/m2 for 12 weeks) either alone or in combination with nintedanib and from 85 healthy controls was prospectively obtained and individual CSTs and average telomere lenght were determined by HT Q-FISH (high-throughput quantitative FISH). Toxicity was graded according to NCI common toxicity criteria for adverse events (NCI CTCAE V.4.0). The variable under study was “number of toxic episodes” during the 12 weeks of therapy.

The percentage of CSTs ranged from 6.5%–49.4% and was directly associated with the number of toxic events (R^2^ = 0.333; *P* < 0.001). According to a linear regression model, each 18% increase in the percentage of CSTs was associated to one additional toxic episode during the paclitaxel cycles; this effect was independent of the age or treatment arm. Patients in the upper quartile (> 21.9% CSTs) had 2-fold higher number of neuropathy (*P* = 0.04) or fatigue (*P* = 0.019) episodes and >3-fold higher number of myalgia episodes (*P* = 0.005). The average telomere length was unrelated to the incidence of side effects.

The percentage of CSTs, but not the average telomere size, is associated with weekly paclitaxel-derived toxicity.

## INTRODUCTION

Weekly paclitaxel is a commonly administered cancer chemotherapy regimen in breast cancer and other malignancies due to its efficacy and tolerability both in early and advanced disease [1, 2]. Toxicities include peripheral sensory neuropathy, fatigue and myalgia. Less frequently, nausea, vomiting, anemia, neutropenia and mucositis/diarrhea are observed [1, 2]. It is not unusual to withold or interrupt paclitaxel because of non-tolerable neuropathy, fatigue or myalgia while patients are still experiencing clinical benefit, due to cumulative dose and interaction with previously administered neurotoxic drugs, which may affect the overall efficacy of the drug. Early detection of those patients at high risk of developing toxic complications, as well as understanding the physiopathology behind the toxicity, may help to perform personalized treatment decisions and develop supportive care alternatives.

Telomere shortening is observed in aging human and most eukaryotes, and it is related to the limited proliferative capacity of tissues such as those targeted by chemotherapy [3, 4]. Aging is associated with higher toxicity of cancer chemotherapy agents even when adjusting by the existing comorbidities that are observed in older patients [5, 6]. The ultimate physiological changes and causes underlying the phenotype of “aging” are not fully understood but involve nine hallmarks, reviewed elsewhere [7]. One of those hallmarks is telomere attrition. Telomeres shorter than 3 kilobases (critically short telomeres, CSTs) can't be repaired by any of the known DNA-repair mechanisms, leading to a chronic DNA-repair response causing apoptosis [8, 9], which seems to be a major cause of limited tissue regenerative capacity. Average telomere length has been reported to associete with chemotherapy toxicity, but the degree of relationship is unclear [10, 11]. We and others have proposed that the percentage of CSTs, rather than average telomere length, is a more accurate determinant of the “biological age” and global cell and tissue dysfunction [12, 13]. Until recently, determining the percentage of critically short telomeres had a low throughput using the available techniques. We have recently solved this by developing an automated high-throughput quantitative telomere FISH platform (HT Q-FISH)[14]. Thus, we propose that, by using CSTs, a population more vulnerable to the side effects of paclitaxel might be detected early and it could be a target for potentially “resetting” to a fitter phenotype in the future. Telomere parameters, according to preclinical and clinical data, are modifiable through telomerase activation or danazol [15–18]. This feature is in contrast with the genetic polymorphisms associated with paclitaxel toxicity in previous studies [reviewed elsewhere [19], that would complicate potential interventions.

We sought to study the value of CSTs in predicting toxicity in treatment-naive patients exposed to weekly paclitaxel in a controlled setting: a randomized clinical trial (CNIO-BR-003/GEICAM-2010/10) with accurate toxicity monitoring and grading according to the NCI common toxicity criteria for adverse events (NCI-CTCAE) V.4.0[20].

## RESULTS

### Patients and controls: clinical characteristics, telomere length, efficacy and toxicity data

From July 2012 to November 2013, 130 patients were recruited in 15 sites in the CNIO-BR-003/GEICAM 2010/10 trial (NCT 01484080). Of those, 115 patients (88.5%) participated in the telomere sub-study and had an adequate sample for analysis. There were not significant clinical or demographic differences between the patients that were valid for analysis and the patients that did not participate in the telomere substudy (13 because of not signing informed consent or having an inadequate sample and 2 because of screening failure and lack of toxicity data availability) (Table 1).

**Table 1:** Demographic and clinical characteristics of patients and controls

Characteristic	Clinical trial patients			Controls	*P* value*
	Participated in telomere substudy (*N* = 115; 88.5%)	Did not participate in telomere substudy (*N* = 15; 11.5%)	All Patients (*N* = 130)	*N* = 85	
**Age (median, range)**	47.5 (30.6–81.4)	48.9 (32.7–72.3)	47.6 (30.6–81.4)	44.8 (20.0–82.9)	0.90
**ECOG PS** **0** **1**	113 (98.3%) 2 (1.7%)	15 (100%) 0 (0%)	128 (98.5%) 2 (1.5%)	85 (100%) 0 (0%)	0.91
**Study arm** **Exp**. **Std**.	57 (49.5%) 58 (50.5%)	8 (53.3%) 7 (46.7%)	65 (50%) 65 (50%)	N/A	N/A
**cT** **T2** **T3** **T4**	84 (73.0%) 29 (25.2%) 2 (1.7%)	10 (66.6%) 3 (20.0%) 2 (14.3%)	94 (72.3%) 32 (24.6%) 4 (3.0%)	N/A	N/A
**cN** **N0** **N1** **N2** **N3**	57 (49.5%) 52 (45.2%) 5 (4.3%) 1 (0.8%)	7 (46.6%) 6 (40.0%) 2 (13.3%) 0 (0%)	64 (49.2%) 58 (44.6%) 7 (5.3%) 1 (0.8%)	N/A	N/A
**Hormonal receptors** **Positive** **Negative**	90 (77.5%) 25 (22.3%)	12 (80.0%) 3 (20.0%)	102 (75.1%) 28 (21.9%)	N/A	N/A

^*^P value: comparisons made between controls and 115 patients valid for analysis.

The age and ECOG performance status of the healthy volunteers (two variables potentially related with toxicity) were not statistically different to the patients in the trial (Table 1).

**Figure 1:**
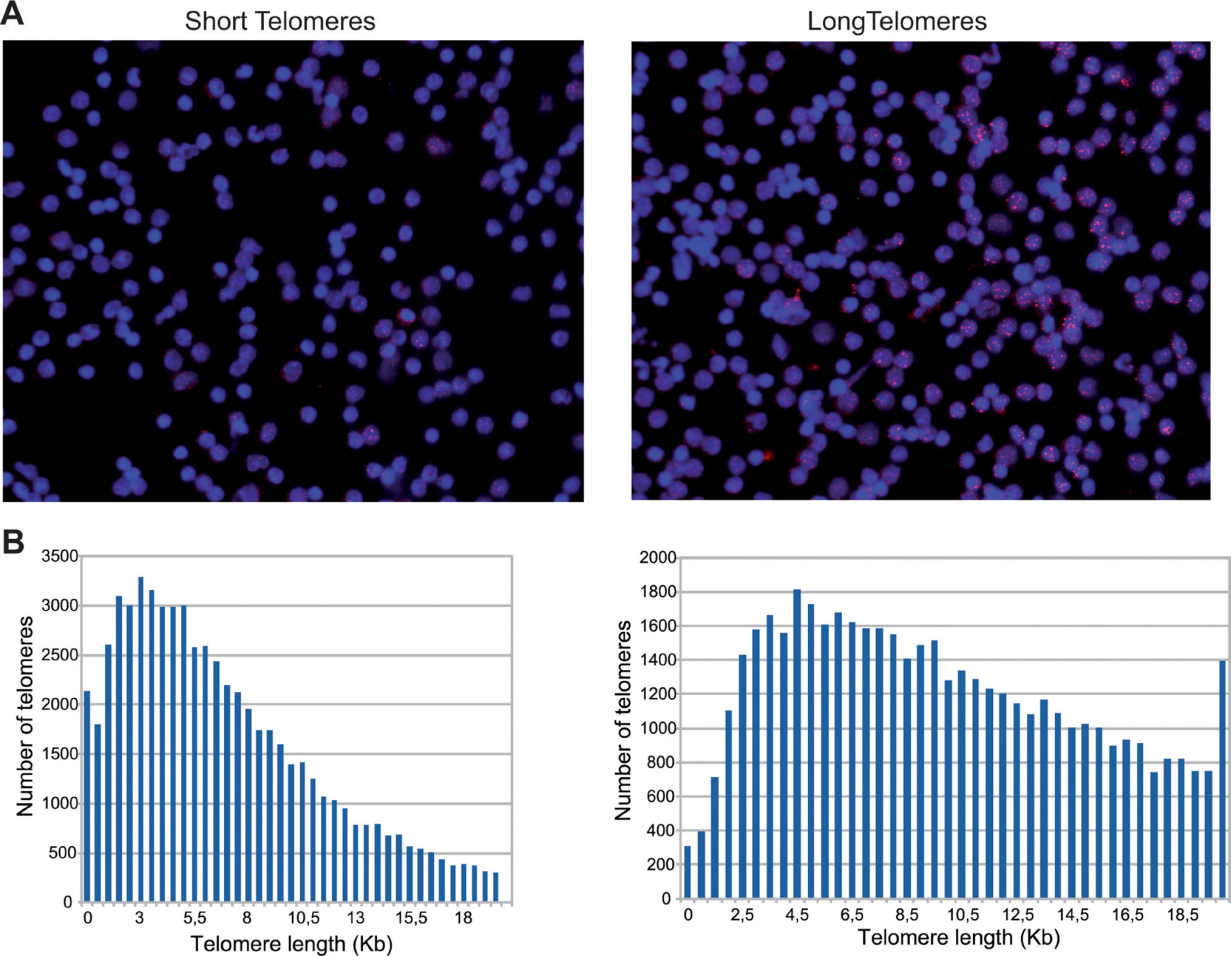
(**A**) HT Q-FISH: pictures from a patient with most telomeres below 3KB (left) and a patient with most telomeres above 3 KB (right) . (**B**) Histograms depicting the telomere determinations from patients shown in (A). Each bar represents the number of telomeres determined within 2 telomere lengths in 0.5 kilobase-increments per sample. The number of telomeres measured per sample is greater than 60000.

**Figure 2:**
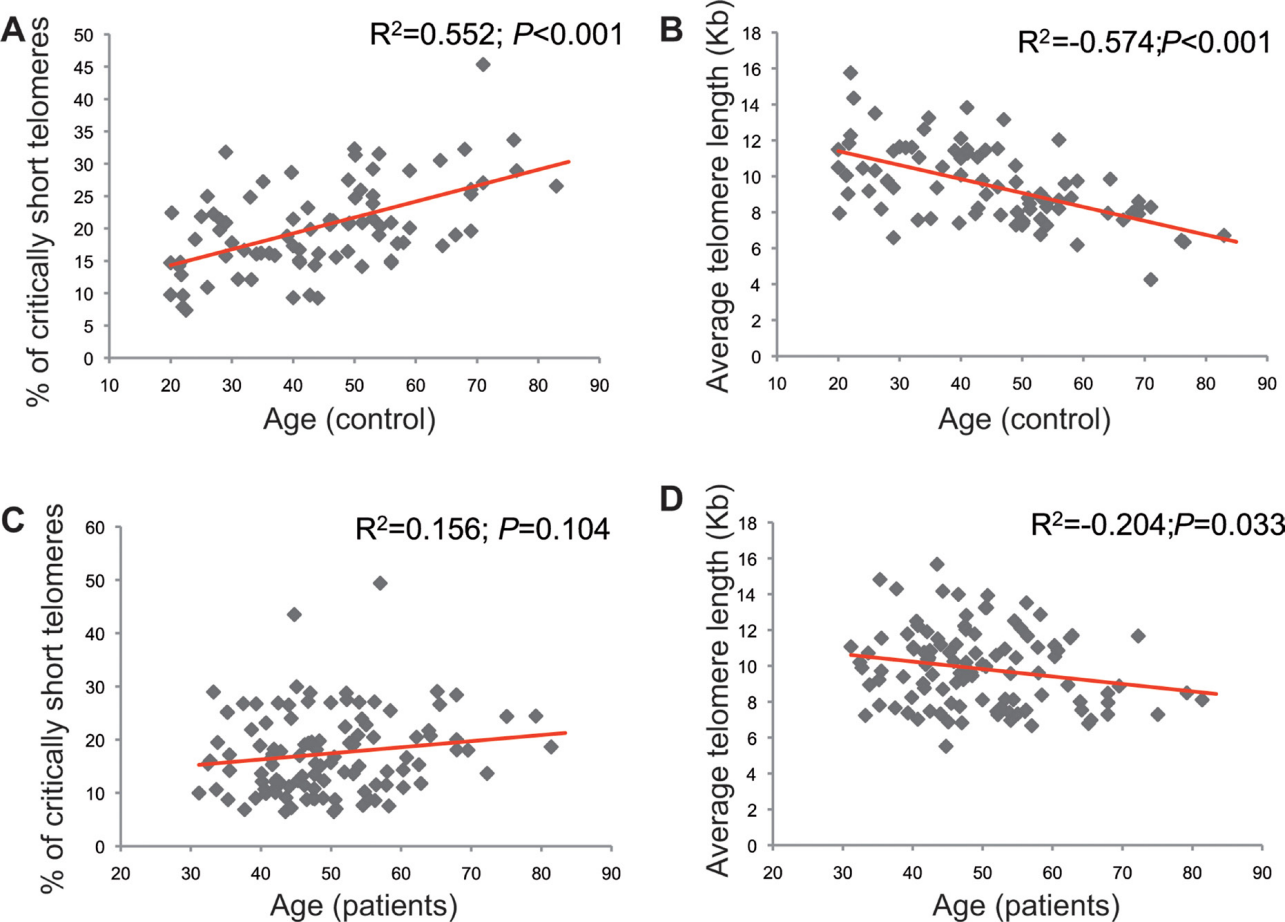
Correlations between telomeric parameters and age in controls (**A**, **B**) and patients (**C**, **D**). The charts in the left (A, C) correspond to the correlation between critically short telomeres and age, whereas the charts in the right (B, D) depict the correlation between the individual average telomere length and age.

The percentage of CSTs in the study patients was 17.4% and the average telomere length was 9.85 Kb. In the healthy volunteers cohort, the percentage of CSTs was 20.5% and the average telomere length was 9.49 Kb. The comparison between the CSTs observed the study patients versus the healthy controls was statistically significant (*p* = 0.004), whereas the comparison of average telomere length between both populations was not (*p* = 0.92). HT Q-FISH examples are shown in Figure 1. In control patients, both the percentage of CSTs and the average telomere showed a good correlation with age. The percentage of CSTs increased with age (R^2^ = 0.552; *P* < 0.001) whereas average telomere length decreased with age (R^2^ = –0.574; *P* < 0.001). A similar pattern was found in the cancer patients, although the intensities of the correlations were less marked (R^2^ = 0.156 and *P* = 0.104 for CSTs, and R^2^ = –0.204 and *P* = 0.033 for average telomere length). The dot plots are shown in Figure 2.

The efficacy and toxicity data of paclitaxel in the study are reported in detail elsewhere [20]. Briefly, the pathologic complete response (pCR) rate was 13.1% in the experimental arm and 11.3% in the standard arm (*P* = 0.61), with a trend towards a higher pCR in the hormone-receptor positive population in the experimental arm [20]. Treatment-related toxicity was similar among the two arms with the exception of neurotoxicity, with an increase in the incidence of this parameter in the standard arm [20]. The paclitaxel-related toxicities under study in this report (peripheral neuropathy, myalgia and fatigue) are described in Table 2. None of them reached grade 3/4; thus, the analysis is limited to grade 1 and 2, which are, in turn, the most frequent toxic events with this drug [1, 2].

**Table 2:** Paclitaxel-derived toxicities in the two study arms

	Arm A (paclitaxel plus nintedanib)	Arm B (paclitaxel)	*P* value
**Grade 1 or 2**			
**Neuropathy (average, range)**	0.6 (0–3)	1.1 (0–4)	0.023
**Myalgia (average, range)**	0.3 (0–3)	0.35 (0–3)	0.689
**Fatigue (average, range)**	1.4 (0–4)	1.6 (0–4)	0.38
	**Arm A (paclitaxel plus nintedanib)**	**Arm B (paclitaxel)**	***P*** **value**
**Grade 3 or 4**			
**Neuropathy (average, range)**	0 (0–0)	0 (0–0)	N/A
**Myalgia (average, range)**	0 (0–0)	0 (0–0)	N/A
**Fatigue (average, range)**	0 (0–0)	0 (0–0)	N/A

### Relationship between telomere parameters, toxicity and response

Patients with a high percentage of CSTs were more likely to experience paclitaxel-related toxicity than those with lower percentage of CSTs. The individual percentage of CSTs ranged, among the 115 patients, from 6.5% to 49.4%. The 75^th^ percentile was 21.9%. Twenty-nine patients (25% of the 115) had greater than 21.9% of CSTs.

**Figure 3:**
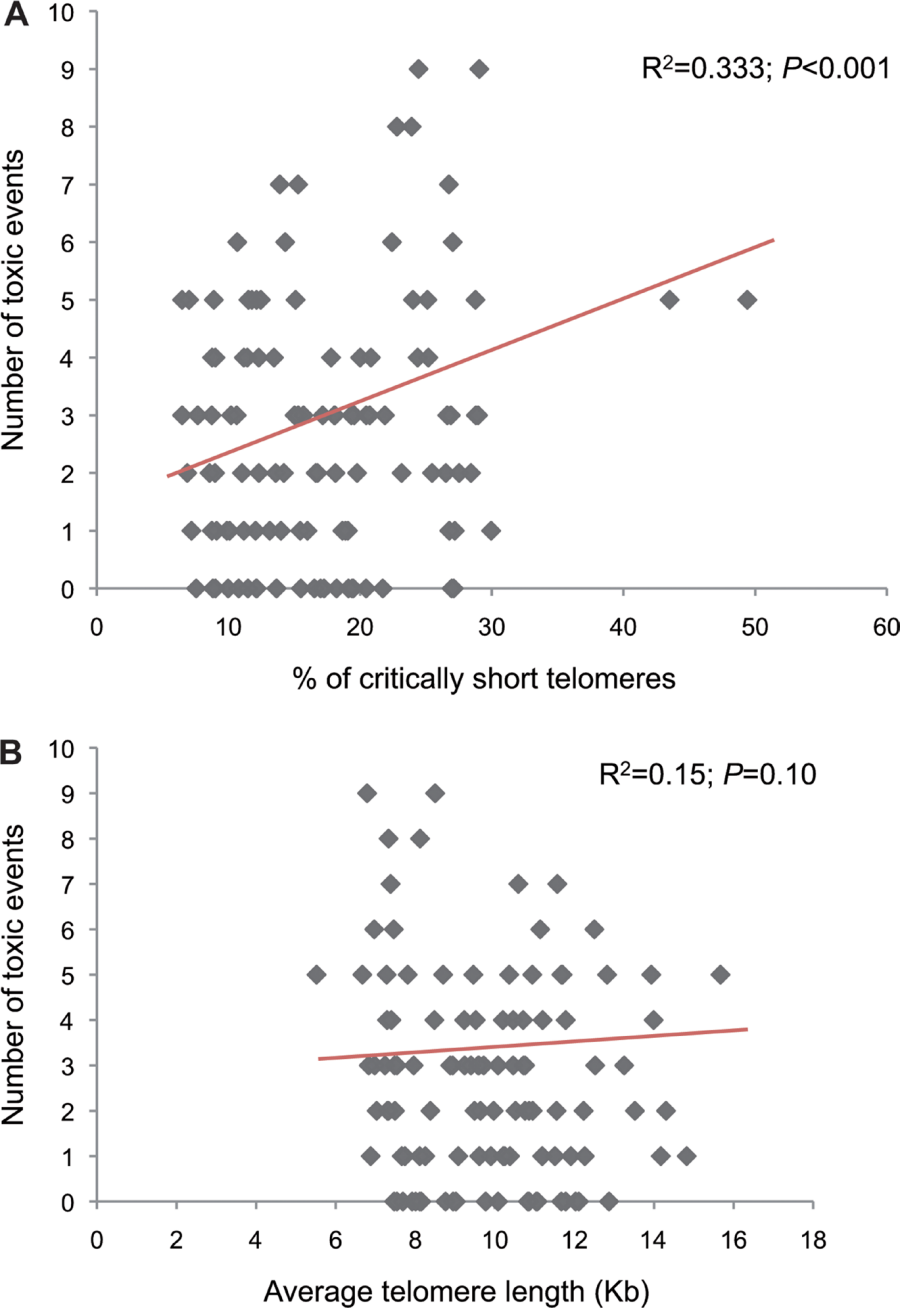
Correlations between the percentage of criticaly short telomeres (**A**) or individual average telomere length (**B**) and number of toxic events.

There was a statistically significant correlation between the percentage of CSTs and the number of toxic episodes derived from paclitaxel administration (Pearson´s R^2^ = 0.333, *P* < 0.001; Figure 3A). When adjusted by age and treatment arm, the linear regression model suggests a quantitative relationship between the percentage of CSTs and the incidence of toxic episodes (B coefficient for percentage of short telomeres = 0.055, *P* = 0.046; the interpretation of this coefficient value would be that per each 18% increase in the percentage of CSTs a patient would experience 1 more paclitaxel-related events along a 12 weeks-treatment course regardless of the age and treatment arm; the B coefficient for age was borderline significant-1 additional toxic event per each 27 years-increment but in this case, because of the lack of significance, it can not be stated that the increased toxicity would be independent of the status of the telomere variable and treatment arm; *P* = 0.069).

The patients in the upper quartile (i.e., highest percentage of critically short telomeres) had a significantly higher incidence of grade 1/2 myalgia (*P* = 0.005), grade 1/2 peripheral neuropathy (*P* = 0.04), grade 1/2 fatigue (*P* = 0.019) or any grade 1/2 toxic events related with paclitaxel (*P <* 0.001). The number of toxic events split by belonging to the upper quartile of critically short telomeres or not is shown in Table 3.

**Table 3:** Average number of cycles where paclitaxel-derived grade 1/2 toxicites were registered according to the percentage of short telomeres or average telomere length

	Average (range) G1/2 episodes		
Toxicity type	Patients with critically short telomeres (*N* = 29)*	Patients without critically short telomeres (*N* = 86)^*^	*P* value (*T*-test)
**Any**	4.0 (0–9)	2.2 (0–7)	< 0.001
**Myalgia**	0.6 (0–3)	0.17 (0–3)	0.005
**Peripheral neuropathy**	1.2 (0–4)	0.7 (0–3)	0.04
**Fatigue**	2.2 (0–4)	1.4 (0–4)	0.019
	**Patients with average short telomere length (*N*** = 29)^**^*	**Patients with average long telomere length (*N*** = 86)^***^*	
**Any**	3.3 (0–9)	2.5 (0–7)	0.100
**Myalgia**	0.21 (0.3)	0.30 (0–3)	0.55
**Peripheral neuropathy**	0.57 (0–4)	0.4 (0–3)	0.135
**Fatigue**	1.9 (0–4)	1.3 (0–4)	0.163

^*^Patients with critically short telomeres: patients in whom > 21.9% of their telomeres were shorter than 3 Kb.

^**^Patients without critically short telomeres: patients in whom < 21.9% of their telomeres were shorter than 3 Kb.

^***^Patients with average short telomeres: in this case, the quartile of patients is selected on the basis of the telomere length distribution. One quartile is still 29 patients (25% of 115 patients); however, the 29 patients defined by the upper quartile of critically short telomeres are not the same patients as those 29 (one quartile) defined by average short telomeres. The cut-off point for average long vs. short telomeres is 8 Kb.

^****^Patients with average long telomeres: patients whose average telomere length is > 8 Kb. Similarly to the previous paragraph, these patients are not the same as those 86 patients defined by having few critically short telomeres.

The individual average telomere length ranged from 5.5 to 15.7 Kb. The 25th percentile was 8.0Kb. We did not find a statistically significant correlation between the individual average telomere length and the number of toxic episodes (Pearson´s R^2^ = 0.15; *P* = 0.100; Figure 3B). The linear regression predicting the number of toxic episodes according to the average telomere length and age preserved a borderline statistically significant association with age (1 additional toxic event per each 25.6 years-increment; B = 0.039, *P* = 0.05), but not with average telomere length (B = –0.077, *P* = 0.338). The patients in the lower quartile (average telomere length short) showed some trend towards association with paclitaxel-derived toxicity, but none of the associations was statistically significant (Table 3).

Because of the lower incidence of neuropathy in the experimental arm, we also adjusted the model by receiving or not nintedanib. The impact of being allocated to the experimental or standard arm. The 25th, 50th, and 75th percentile of the CSTs were very similar between the experimental (11.2%, 15.6% and 21%) and standard (11.4%, 16.8% and 24.2%) arms (*P* = 0.91); thus it is not likely that the observed differences in neuropathy were observed because of different CSTs distribution between arms. The multivariate analysis (adjusted by CSTs, age, and treatment arm) shows a borderline statistically significant trend towards toxicity protection in the standard arm (B coefficient = 0.68; *P* = 0.10; the interpretation would be that regardless of the CSTs status and age, a patient would have a non-statistically significant decrease in the risk of paclitaxel-derived toxicity if he/she received nintedanib as well).

We also explored the univariate relationship between “numerical age” and the incidence of toxicity. Age was not an individual predictor of toxicity (Table 1). The 29 patiets with an age above the upper quartile (patients above 56.06 years-old) had a similar number of paclitaxel-related side effects (average *N* = 3.26 episodes) than those patients with younger ages (patients below 56.06 years, 86 patients; average *N* = 3.25 episodes; *P* = 0.981). When the side effects were analyzed by class [myalgia (0.67 versus 0.89 episodes for older versus younger; *P* = 0.365), neuropathy (0.90 versus 0.84 episodes for older versus younger; *P* = 0.79); or fatigue (1.52 versus 1.65 episodes for older versus younger; *P* = 0.65)] no significant differences were found either.

None of the main toxic events related with nintedanib (transaminase elevation, hypertension, hand-foot syndrome or diarrhea) [22] correlated with the percentage of short telomeres nor the average telomere length, although the incidende of these events in our series was low [20]. Telomeric status was no associated with tumor response either: according to the Symmnans and Pusztai classification for pCR [23], 14.3% of the patients with CSTs in the upper quartile experienced a response of 0 or 1, compared with 16.2% of the remaning patients (*P* = 0.93).

## DISCUSSION

Both the increasing number of cancer survivors and the prolonged survival of patients with advanced disease have lead to an increasing number of patients with chronic and sometimes irreversible toxicities. Weekly paclitaxel is one of the most commonly administered cytotoxic agents in many different cancer types and it is related with several toxicities that can both limit its use and have a chronic impact in the patient´s quality of life, despite its high activity [24]. Thus, it is highly prioritary to define whether there are factors associated with cummulative toxicity observed with this treatment regimen and understand its physiopathology, and to develop potential solutions in the patients most likely to have their paclitaxel dosing interrupted because of toxicity. This latter point is important, since other studies investigating potential factors involved in paclitaxel toxicity [reviewed elsewhere [19] focused in non-modifiable genetic traits.

We present the data from a molecular sub-study that determined telomere length in the CNIO-BR-003/GEICAM-10/10 clinical trial, in which early HER2-negative breast cancer patients received single-agent weekly paclitaxel (standard arm) or weekly paclitaxel plus nintedanib. We report that the load of CSTs, but not average telomere length, predict an almost two-fold incidence of the side effects most commonly associated to paclitaxel.

Telomere fitness is associated with the ability of the stem cells to repopulate tissues (“tissue regeneration”), what would make the recovery of toxicities a longer and more complicated process, and ultimately, more evident for the patients and clinicians. However, preclinical research suggests that it is the burden of CSTs, and not the individual average telomere length, what represents a limiting factor for maintaining tissue homeostasis [8, 9, 12, 13]. Thus, not surprisingly, the associations between telomere length and chemoterapy-related toxicity are unclear [10, 11]. We did not find associations between the individual average telomere length and toxicity (Table 3, Figure 3B).

The determination of CSTs in a high throughput and accurate manner represented an important challenge due to various factors, reviewed elsewhere [12]. We developed an automated, high-throughput technique to overcome the existing limitations (HT Q FISH)[14], and we explored the association of this parameter with paclitaxel-derived toxicity in parallel with the average telomere length. The patients with the highest percentage of critically short telomeres were almost two-fold more likely to experience fatigue, myalgia and neuropathy (Table 3). The total number of toxic episodes was correlated with the percentage of critically short telomeres (Figure 3A). This correlation was independent of the age effect, as evidenced by the linear regression model. While age has been traditionally associated with toxicity from chemotherapy in breast cancer [5, 25], “age” is a complex phenotypical trait that translates many underlying biological factors, one of which is telomere attrition [7]. As our understanding of the aging phenotype advances, we will be able to finely pinpoint the relative influence of each of the underlying biological factors (which, in turn, represent the “biological age”). Telomere attrition seems to be one major contributor to the aging phenotype, but, at least in the case of toxicity prediction, it seems to overperfom the “numerical” patient age. Not surprisingly, the correlation between the status of telomeres and the numerical age is, at best, modest (Figure 2A–2D). As it can be appreciated in this figure, patients/healthy volunteers with the same numerical age can differ up to three-fold in the percentage of critically short telomeres or in the average telomere length. Previous studies, reviewed elsewhere [5], show an association between age and taxane-derived toxicity in breast cancer; however, those studies required large number of patients to show the association. Numerical age, by itself, was unable to predict toxicity in our study, as opposed to CSTs, suggesting that CSTs is a variable that more accurately reflects the true biological age than the numerical age. Cancer incidence seems to increase with age; however, the relationship with telomere length is less clear [26–34]. In case the incidence was higher in patients with short telomeres, it could as well imply that cancer patients will be more prone to toxicity than the general population. However, our results suggest an even fitter telomere status for the patients than the control subjects (statistically significant smaller percentage of critically short telomeres). A similar observation was made by Svenson and colleagues, who detected significant differences between breast cancer patients and control subjects in telomere length, favoring the former [35]. Despite telomerase activity in cancer cells, cancer cell telomeres are usually shorter than in the corresponding normal tissue [36]. Taken together, these data suggest a complex relationship between telomere fitness, numerical age and cancer/toxicity development. Fitter telomeres would favor cancer development and protect from chemotherapy-derived toxicity; the role of telomere fitness in cancer cells and response to treatment reminds to be elucidated. Finally, besides age, several genetic and environmental factors influence telomere shortening; our control and study populations might have had different exposure to such factors what would account for the observed differences, but those data were not gathered in our study [37, 38]. All these scenarios should be carefully assessed in independent studies.

The strengths of this study are that it was a prospectively, pre-planned study and that a high percentage (88.5%) of the trial participants donated a blood sample for telomere determinations. Toxicity data were recorded within a clinical trial according to NCI CTC AE criteria. In addition, this study was performed in chemotherapy-naive patients and the patients received only one cytotoxic agent; other studies have found associations with paclitaxel toxicity but in many cases the patients received previous and/or concurrent chemotherapy agents that can confound the interpretations of the studies [19]. HT Q-FISH is a robust and reproducible technique. The limitations are, the relatively low number of patients enrolled, the semi-quantitative nature of toxicity-reporting methods, and that the linear relationship between critically short telomeres and number of toxic events was not adjusted by the total paclitaxel dose, but it is unlikely that this factor played a role since the patients enrolled in the study received greater than 90% of the planned dose-intensity in all cases [20]. In addition, it is not possible to estimate whether the relationship between critically short telomeres and toxicity would be maintained with severe toxicity as well, since we did not observe grade 3 or 4 paclitaxel-related side effects. In any case, it is very common to have to interrupt chronic paclitaxel administration because of chronic, non-tolerable grade 2 toxicity (fatigue, neuropathy), since the incidence of grade 3/4 events is low. Finally, there may be potentially confounding effects of nintedanib, as the imbalance in the incidence of neurotoxicity across the trial arms could affect the interpretation of the trial results, but according to the multivariate linear regression model, the impact of the percentage of critically short telomeres was independent of the study arm. In any case, our study aimed to assess the association between CSTs and paclitaxel-related toxicity and thus the results would be applicable only to patients receiving single-agent paclitael. Whether and why nintedanib protects from neuropathy requieres confirmation and further research.

This is the first study that performs both telomeric determinations in parallel. Our results, together with the preclinical evidence, might suggest that the percentage of critically short telomeres, but not average telomere length could be used as a toxicity predictive factor. Telomere attrition is a hallmark of aging [7], but recent preclinical observations suggest that it could be a modifiable characteristic. Several genetic approaches have been succesfully used in *in vivo* models [15–17]. Also, male sex hormones up-regulate telomerase enzymatic activity [39], what was used in an *in vivo* model of telomerase dysfunction to improve the hematologic function [40]. This latter approach has been used in a clinical trial as well, where patients with telomere diseases received the synthetic steroid drug danazol; this intervention led to telomere elongation [18]. Danazol up-regulates TERT expression through an estrogen-responsive element in the gene promoter [39, 41]. Before conducting an intervention trial wiht danazol in breast cancer patients receiving taxanes aimed to elongate telomeres in healthy tissues target for toxicity, potential effects of danazol in the cancer cells interfering with chemotherapy efficacy should be studies and taken into account. Although we did not find associations between telomeric condition and treatment efficacy, what could suggest that the effects of an intervention like danazol could be beneficial regarding toxicity without compromising efficacy, this point must be validated prospectively. Thus, measuring the percentage of short telomeres to those patients that are going to receive chemotherapy might help undertaking personalized treatment decisions.

## MATERIALS AND METHODS

### Patients, controls and samples

The CNIO-BR-003/GEICAM-10/10 clinical trial randomized 130 early HER2-negative breast cancer patients to neoadjuvant weekly paclitaxel (80 mg/m2) for twelve courses before surgery or the same schedule plus nintedanib, administered at 150 mg/bid. Patients signing informed consent for the telomere ancillary study were drawn a 7.5-ml peripheral blood sample during the trial screening phase, prior to administration of the first treatment dose. Briefly, the inclusion criteria included female, ≥ 18 year-old patients with histopathologically proven HER2-negative resectable breast cancer larger than 20 mm on its longer diameter, without previous diagnosis of cancer or chemotherapy treatment. Patients with serious comorbidities, or ECOG 2 or higher were excluded as well. Patients were evaluated every 2 weeks and toxicity was clinically assessed according to the NCI-CTC-AE V.4.0. For control purposes, blood samples from a cohort of 85 female volunteers without previous history of cancer were included in the study.

The CNIO-BR-003-GEICAM/2010-10 trial was registered atClinicaltrials.gov (NCT01484080). All study procedures were conducted in accordance with the Declaration of Helsinki and Good Clinical Practice standards. Institutional review board approval was obtained from all participating hospitals and CNIO.

### Telomere length

Samples were collected in CPT tubes (BD Bioscience), which maintain peripheral-blood mononuclear cells (PBMCs) viability for up to 48 hours at room temperature. The samples were shipped to CNIO within < 24 hours and PBMCs were isolated and frozen until analysis, according to manufacturer instructions. High-throughput quantitative fluorescence *in situ* hybridization (HT Q-FISH) with automated fluorescence microscopy was performed as previously described [14]. Briefly, PBMCs were counted and plated (80 000–100 000 cells/well) in clear-bottomed black-well 96-well plates. DAPI (4′,6-diamino-2-phenylindole) was used for nucleus staining and a fluorescent peptide nucleic acid (PNA) Cy3 probe against telomeric repeats was used for telomere detection. Telomere length values were analyzed using individual telomere spots. Fluorescence intensities were converted into Kb using L5178-R, L5178-S and CCRF-CEM cells as calibration standards, which have stable telomere lengths of 79.7 Kb, 10.3Kb and 7.5 kb, respectively [21]. Samples were analyzed in duplicate, or triplicate in the case of calibration standards. A telomere length < 3 Kb was defined as critically short [8, 9]. The load of short telomeres was estimated as the percentage of short telomeres (number of short telomeres divided by total number of measured telomeres) in each participant. Average telomere length was calculated for each patient by adding the length (in Kb) of each measured telomere and dividing that value by the number of measured telomeres.

### Toxicity

The variable under study was “number of toxic episodes”. One toxic episode was defined as the registration of grade 1 to 5 toxicity (according to NCI-CTC-AE V.4.0) at any moment of one three weeks-cycle. Since most of the adverse events that were observed in the trial were grade 1 or 2, for analytic purposes the toxic episodes were defined as “mild” when the grade was 1 or 2, and severe when the grade was 3 to 5. The pre-planned analysis included the three most common toxicities registered with weekly paclitaxel (myalgia, peripheral neuropathy and fatigue), although an exploratory analysis was performed with the less frequent toxicities related to paclitaxel or those related to nintedanib as well [2, 22].

### Statistical considerations

T-tests were used to compare average values in independent groups, whereas Z-tests were used to compare percentages. Correlations between variables were explored with the Pearson's test. A linear regression model was used in order to explore the influence of telomere length or the percentage of critically short telomeres in the number of observed toxic events (from 0 to 12) during the 4 cycles, adjusted by age and treatment arm. The pathologic complete response (pCR) categories were compared with the Chi-square according to the CSTs status. All analyses were performed with the SPSS v.19 software.

## Abbreviations

CSTs: critically short telomeres
HT Q-FISH: high-throughput quantitative telomere FISH
NCI CTC AE V.4.0: NCI common toxicity criteria for adverse events V.4.0
PBMCs: peripheral-blood mononuclear cells
DAPI: 4′,6-diamino-2-phenylindole
PNA: peptide nucleic acid
pCR: pathologic complete response.
